# Effective Stiffness of Fused Deposition Modeling Infill Lattice Patterns Made of PLA-Wood Material

**DOI:** 10.3390/polym14020337

**Published:** 2022-01-15

**Authors:** Enrique Cuan-Urquizo, Alberto Álvarez-Trejo, Andrés Robles Gil, Viridiana Tejada-Ortigoza, Carmita Camposeco-Negrete, Esmeralda Uribe-Lam, Cecilia D. Treviño-Quintanilla

**Affiliations:** 1 School of Engineering and Science, Tecnologico de Monterrey, Querétaro 76130, Mexico; ecuanurqui@tec.mx (E.C.-U.); A01205044@itesm.mx (A.Á.-T.); A01704315@itesm.mx (A.R.G.); viri.tejada@tec.mx (V.T.-O.); 2 Laboratorio Nacional de Manufactura Aditiva y Digital (MADIT), Autopista al Aeropuerto, Km. 9.5, Calle Alianza Norte 100, Apodaca 66629, Mexico; 3 School of Engineering and Science, Tecnologico de Monterrey, Toluca 50110, Mexico; carmitacamposeco@tec.mx

**Keywords:** additive manufacturing, infill, fused deposition modeling, 3-point bending, lattice

## Abstract

Fused deposition modeling (FDM) uses lattice arrangements, known as infill, within the fabricated part. The mechanical properties of parts fabricated via FDM are dependent on these infill patterns, which make their study of great relevance. One of the advantages of FDM is the wide range of materials that can be employed using this technology. Among these, polylactic acid (PLA)-wood has been recently gaining attention as it has become commercially available. In this work, the stiffness of two different lattice structures fabricated from PLA-wood material using FDM are studied: hexagonal and star. Rectangular samples with four different infill densities made of PLA-wood material were fabricated via FDM. Samples were subjected to 3-point bending to characterize the effective stiffness and their sensitivity to shear deformation. Lattice beams proved to be more sensitive to shear deformations, as including the contribution of shear in the apparent stiffness of these arrangements leads to more accurate results. This was evaluated by comparing the effective Young’s modulus characterized from 3-point bending using equations with and without shear inclusion. A longer separation between supports yielded closer results between both models (~41% for the longest separation tested). The effective stiffness as a function of the infill density of both topologies showed similar trends. However, the maximum difference obtained at low densities was the hexagonal topology that was ~60% stiffer, while the lowest difference was obtained at higher densities (star topology being stiffer by ~20%). Results for stiffness of PLA-wood samples were scattered. This was attributed to the defects at the lattice element level inherent to the material employed in this study, confirmed via micro-characterization.

## 1. Introduction

In the optimization of weight without compromising mechanical properties, nature has made use of porous materials [1]. This has inspired engineers and designers to explore the use of tessellated arrangements of matter, known as *lattice* materials [2,3]. Structures with an engineered distribution of material have various advantages when compared to their fully-dense or quasi-solid counterparts [3]. In some additive manufacturing (AM) processes, this arrangement appears naturally [4]. Fused deposition modeling (FDM) or fused filament fabrication (FFF) makes use of a tessellated distribution of rasters known as *infill*. Each layer in the FDM process consists of contour rasters and infill patterns. While this leads to savings in materials and reduction in weight of the AM component, it also affects their resulting mechanical properties [5].

The trajectories of the extruder in FDM machines are generated via slicing software where two main infill parameters can be modified: (i) pattern (topology) and (ii) infill density $\bar{\rho }$. The infill density (or percentage) is related to the relative density (volume fraction). However, there can be differences between these two as there is material overlapping in certain regions of the 3D printed part. Currently, there is no slicing software that provides the user with an insight on what to expect in terms of mechanical properties upon the selection of parameters (i) and (ii). Recently the effect of infill on the mechanical properties of FDM parts has been studied [6,7,8]. Kiendl and Gao [6] characterized how the rasters orientations in FDM affect the stiffness, strength, and toughness of tensile samples. Anisotropy was more evident for toughness with rasters in layers oriented at alternating 90°. Layers with alternating 90° rasters were also studied by Aloyaydi et al. [7] but for FDM samples under 3-point bending. Four different infill densities were tested 40, 60, 80, and 100%. Concentric, square, honeycomb and Hilbert curve infill topologies were studied by Akhoundi and Behravesh [8] under tension and bending. The influence of the topology was more evident at low relative infill densities (20 and 50%).

Infill patterns make use of lattice materials, which have been studied extensively [9]. The effective or apparent properties of these depend on the relative density and the topology [10]. Hence, the mechanical properties of FDM parts depend on these two, but they can also be affected by printing orientation, layer thickness, and material properties, among others [11]. Not only does the mechanical performance depend on these, but the printing quality is also directly affected [12]. Another phenomenon that affects the mechanical performances of FDM parts is the crystallization, influencing the bonding or welding between layers [13].

One of the advantages of FDM is the variety of materials that can be printed. The most popular are polylactic acid (PLA) [14,15] and acrylonitrile butadiene styrene (ABS) [16,17]. Recently, the range of materials for FDM has been expanded with other materials, such as thermoplastic polyurethane (NinjaFlex) [18], polycarbonate [19,20], polyvinyl alcohol [21,22], and PLA-wood [23]. PLA is the most popular material for FDM and has been used as matrix in composites with PLA/wood fibers with the purpose of enhancing the mechanical properties [24], reducing cost, increasing strength, and covering functional load-bearing applications [25]. Additionally, low toxic emissions lead to more environment-friendly conditions of the material [26].

All the possible applications of FDM have driven studies to investigate the effect of the printing parameters on the mechanical properties of PLA/wood composites. Lebedev et al. [27] studied the effect of adding wood flour to PLA on the mechanical properties of FDM printed and hot-pressed samples. Their results showed an increment between 70 and 83% of the Young’s modulus because of the restriction in the polymer mobility and a significant decrease in strength and elongation-at-break because the filler particles act as stress concentrators, diminishing the adhesion between the interfaces. Hot-pressed samples presented higher mechanical properties in comparison with the printed ones owing to the high porosity and poor compaction of filament layers. The interfacial bond strength between layers is a critical parameter that could affect the mechanical properties. As FDM does not involve high molding pressure or high shear rate, as do traditional thermoplastic processes, the material fails to develop high cohesion with strong interlayer interactions [28].

The use of FDM with PLA-wood creates issues that affect the mechanical properties. Recently, the effect of adding cellulose-based fillers to a polymeric matrix on the enhancement of the mechanical properties was reported in [29,30]. A recent study showed that the filling ratio of the printing parts will have a direct effect on the tensile properties of PLA-wood parts, decreasing between 18 and 30% the strength of the printed part [31]. Using composites adds another level of inhomogeneity and anisotropy to the FDM part. Using PLA-wood in FDM leads to delamination among layers which, together with other defects such as high porosity, poor compactation, humidity [32], and bad adhesion between layers, affects the suitability of the printing process [33,34].

Travieso-Rodriguez et al. [35] characterized the mechanical properties of PLA-wood FDM samples subjected to fatigue loading. The inclusion of wood fibers resulted in poor adhesion and increased number of voids between fibers [35]. Stress concentrators resulted from these voids. Travieso-Rodriguez et al. [36] reported a comparative study of the mechanical properties between ABS, PLA, and PLA-wood. The inclusion of wood particles in a PLA matrix could lead to reductions of up to ~35% in stiffness and ~57% strength [36]. Authors in [36] concluded that PLA-wood properties are lower than those of PLA and ABS, and recommended using this material for aesthetic purposes. Among the different manufacturing parameters tested, the infill topology influence on the stiffness and strength was minimal as the samples were printed with contour rasters. Hence, the most influential parameters obtained in [36] were layer height, orientation, and nozzle diameter. Tao et al. [37] studied the effect of the raster width on the compression strength of PLA-wood FDM parts printed using different topologies. The results showed that for wider rasters porosity increases, affecting the compression performance. The properties under tensile loading of PLA-wood structures fabricated via FDM and filled with the square lattice topology were characterized in [38]. For the stiffness, the infill density and the printing orientation proved to be more influential. FDM samples were also compared with those fabricated via injection, and the latter displayed superior properties for both stiffness and strength.

Despite the advantages of lattice materials and AM processes, the characterization of the mechanical properties of PLA-wood AM samples is still an open issue. A recent work where PLA-wood samples (with hexagonal honeycomb infill lattice) were subjected to 4-point bending was published [23]. In [23], the effect of several manufacturing parameters, including layer height, nozzle diameter, infill density, and printing velocity, on the mechanical properties was studied. Zandi et al. [23] reported that the parameters of layer height on the elasticity and nozzle diameter and infill density on the plastic properties showed higher influences, respectively. Despite this, the relevance of the lattice topology to the mechanical properties was not deeply studied. The review of the literature suggests that PLA-wood *lattices* produced via FDM have not yet been characterized mechanically. This motivates the present paper where PLA-wood *lattice* AM samples are subjected to 3-point bending and the effective Young’s and Shear moduli characterized. Additional micro-characterizations of the lattices and the failure areas are included.

## 2. Materials and Methods

### 2.1. PLA-Wood Material for FDM

Wood PLA Filament from Hatchbox^®^ (1.75 mm, print temperature 180–220 °C, Pomona, CA, USA) was used as printing material. According to the supplier specifications, the wood fiber percentage is 11% recycled wood particles. More information can be obtained from the manufacturer’s webpage (Hatchbox PLA Wood—1.75 mm, 1 kg spool, Diameter Tolerance +/− 0.03 mm, 3D Filament—HATCHBOX 3D).

### 2.2. Design and Additive Manufacturing of Lattice Samples

**Figure 1:**
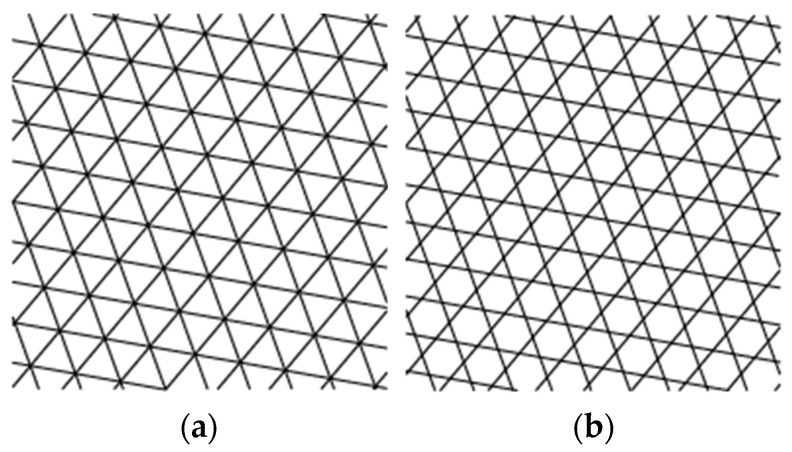
The infill patterns studied: (**a**) hexagonal, (**b**) star.

**Figure 2:**
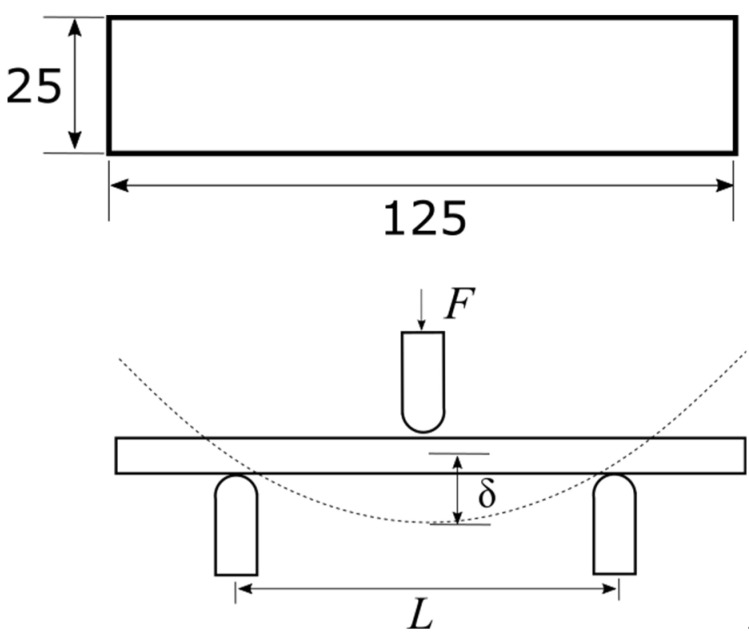
Sample dimensions (mm) and testing schematic; deformed shape is presented with dotted line. Thickness of the samples is 6 mm.

**Figure 3:**
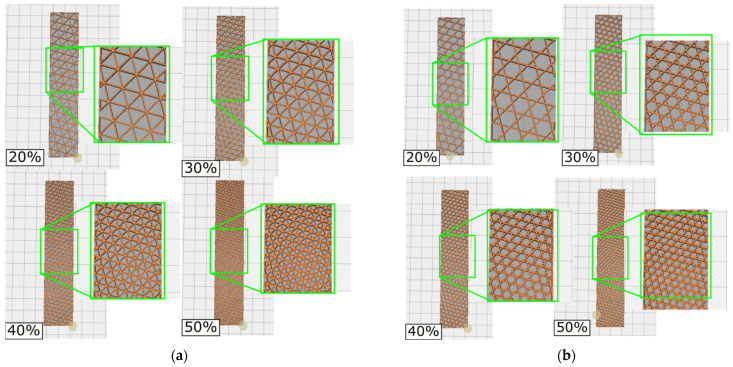
Infill patterns for (**a**) hexagonal and (**b**) star showing the different infill densities.

**Figure 4:**
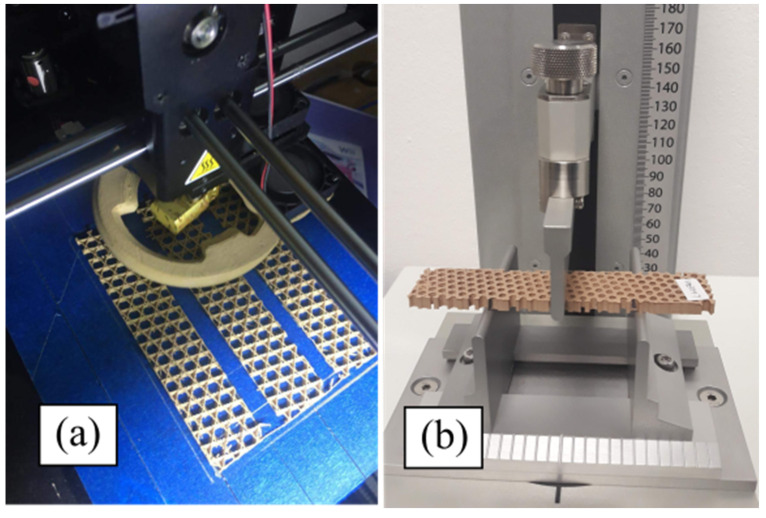
Photograph of (**a**) 3D printing process of lattice infill samples and (**b**) sample mounted on 3-point bending testing setup.

The infill patterns studied in this work are depicted in Figure 1. In order to relate the data from the mechanical testing to the infill parameters, samples were printed with no contour rasters (without outer walls). When contour rasters are parallel to the principal axis of the samples, these participate in the withstanding of the load, reducing the participation of the infill in the loading bearing [11]. Samples were modeled in SolidWorks^®^ (v2019, Dassault Systèmes, Waltham, MA, USA) with the dimensions shown in Figure 2 and then processed in CURA^®^ v4.3.0 (Ultimaker B.V., Utretch, The Netherlands). Samples with different infill densities (20, 30, 40, and 50%) were generated. These infill densities were selected considering two aspects. First, above 50%, the topology is almost lost, as the voids are smaller. Second, below 20%, the number of rasters within the sample are too few (not enough to be considered lattice material). The related literature included works where the mechanical properties of PLA-wood structures were characterized only for two infill topologies: square [38] and honeycomb [35]. Hence, the infill patterns in Figure 1 have not been previously studied under the conditions used here. These infill topologies were triangles (referred here as *Hexagonal*) and tri-hexagon (referred here as *Star*), shown in Figure 3a,b, respectively. The samples were fabricated from PLA-wood material by Hatchbox^®^ using the MP Select Ultimate^®^ (Wanhao D6) FDM-machine (Zhejiang, China). This machine has a building volume capacity of 20 cm × 20 cm × 17.5 cm, a nozzle diameter of 0.4 mm, travel and printing speeds of 30–350 mm/s and 30–300 mm/s, respectively. Samples were fabricated in sets of three, oriented as in Figure 4a. Printing parameters are included in Table 1. From 15 different measurements of randomly selected rasters that conformed the samples, the width of such was obtained as 512.73 ± 34.29 µm (these measurements via micro-characterization as detailed in Section 2.3).

### 2.3. Mechanical Testing and Micro-Characterization

The effective mechanical properties of the infill patterns were characterized via 3-point bending using a Perten^®^ Texture analyzer (Figure 4b, New South Wales, Australia). Triplicates were manufactured for each infill density and tested at three different separations between supports *L* (Figure 2). A total of 72 samples were tested. Dimensions for the samples were obtained from the standard ASTM D790, with a deliberate modification in the width. This was increased to ensure it was significantly greater than the dimensions of rasters forming the lattice. Variables for testing and manufacturing are summarized in Table 1. The different values for *L* were selected taking into consideration the maximum distance between supports attainable with the experimental setup (80 mm) and two lower values. From the software TexCalc^®^ (v5.2.2.287, Perten Instruments, New South Wales, Australia) the data corresponding to force *F* and displacement *δ* were exported to Matlab^®^ (R2021a, MathWorks, Natick, MA, USA) for further processing, similar to the approach presented in [39].

Considering both Young’s *E* and shear *G* moduli, their corresponding contributions to the elastic displacement of a beam simply supported loaded at the midpoint are given by [40]:

(1) $$\delta =F\left[\frac{L^{3}}{48EI}+\frac{L}{4\kappa GA}\right]$$

where *I* is the second moment of area. In this work the effective second moment of area is considered as a rectangle with area *A* (ignoring voids), *κ* is the shear correction factor (5/6 for rectangular cross-sections). Here *A* = 150 mm^2^ and *I* = 450 mm^4^. When shear deformations are ignored, only the first term of Equation (1) is considered. Now, if Equation (1) is rearranged non-dimensionally as

(2) $$\frac{\delta }{L}=\frac{F}{48EI}L^{2}+\frac{F}{4\kappa GA}$$

Plots with *L*^2^ vs. *δ/L* axes result in straight lines from which the slope is *F/48EI* and the intercept *F/4κGA*. From these, the moduli *E* and *G* can be obtained. If such beams are composed of a lattice arrangement, the moduli obtained correspond to their effective properties, i.e., 〈*E*〉 and 〈*G*〉. Hence, the transverse displacement of a lattice beam with effective properties 〈*E*〉 and 〈*G*〉 can be worked from the following expression:

(3) $$\frac{\delta }{L}=\frac{F}{48\langle E\rangle I}L^{2}+\frac{F}{4\kappa \langle G\rangle A}.$$

Here, lattice PLA-wood samples were subjected to 3-point bending using 3 different distances between the supports *L* (60, 70, and 80 mm). Then, from the linear part of the load-displacement curve the value for *δ* was obtained for a force *F* of 4 N. The effective Young’s modulus was obtained using both Equation (3) and the expression that only considers the first term of Equation (1) (no shear). Now, considering the cross-sectional areas of beams under 3-point bending as if they were fully solid (ignoring voids), the only remaining data from the first term of Equation (1) were those corresponding to the effective Young’s modulus. Hence, these were directly obtained using the experimental data for *F* and *δ,* and the controlled parameters of *L* and *I*. Now, for the shear-inclusive analysis, the data measured for *δ* obtained for *F* = 4 N for each value of *L* tested was arranged in a *L*^2^ vs. *δ/L* plot. Again, ignoring the voids in the beam’s cross-sectional area, the values for *A* = 150 mm^2^ and *I* = 450 mm^4^ were used to obtain the elastic moduli (〈*E*〉 and 〈*G*〉). This comparison was included to highlight the relevance of shear inclusion in the deflection of lattice beams.

The testing machine for 3-point bending (Figure 4b) was used under displacement control, setting a maximum displacement of 20 mm at a velocity of 3 mm/s. Then, the samples were micro-characterized to inspect the structure formed and the defects on the rasters. The microstructural characterization was done with an inverted optical microscope (Carl Zeiss, Bucuresti, Romania) with a magnification of 50× and microphotographs taken with an AxioCam (both by ZeissVR^®^, v4.9.1, Carl Zeiss, Bucuresti, Romania).

### 2.4. Statistical Analysis

Results were reported as means ± standard deviation. Mechanical testing and characterization were carried out in triplicate. One-way analysis of variance (ANOVA) and Tukey tests were used for statistical analysis and for differences among means. Regarding the statistical results, the lowercase letters refer to differences among infill densities (50, 40, 30, and 20%) at the same length, and capital letters refer to differences among lengths (60, 70, and 80 mm) at the same infill density. All statistical evaluations were performed using the Minitab Statistical Software^®^ (v19, State College, PA, USA) with a significance level of ≤0.05.

## 3. Results

### 3.1. PLA-Wood Samples under 3-Point Bending

**Figure 5:**
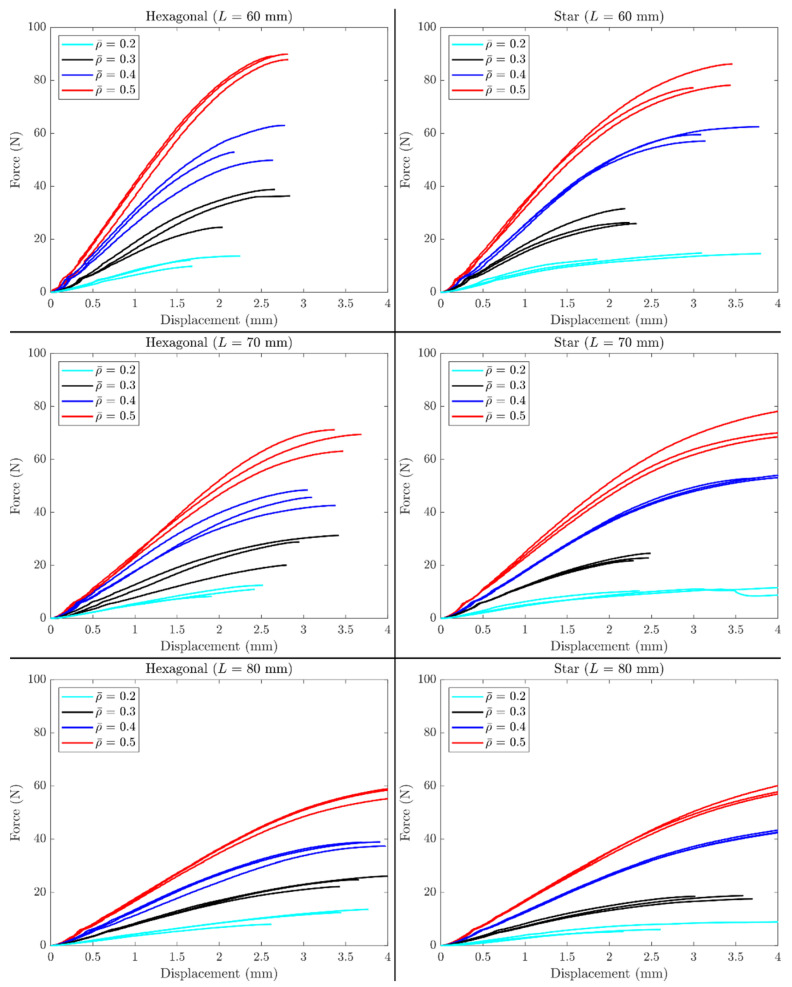
Load-deflection curves for hexagonal structure (**right**), and star (**left**).

Force-deflection curves from tests at three different values of *L* are presented in Figure 5, where the column on the right corresponds to the results of the hexagonal topology and the results of the star topology are presented on the left. Each row corresponds to each value of *L*. As expected, the higher the value of *L*, the softer the structure. An increment in the stiffness and strength of the structures was observed when the infill density of the infill pattern increased. Variability was observed more in strength as this was more sensitive to defects (further shown in Section 3.4).

**Figure 6:**
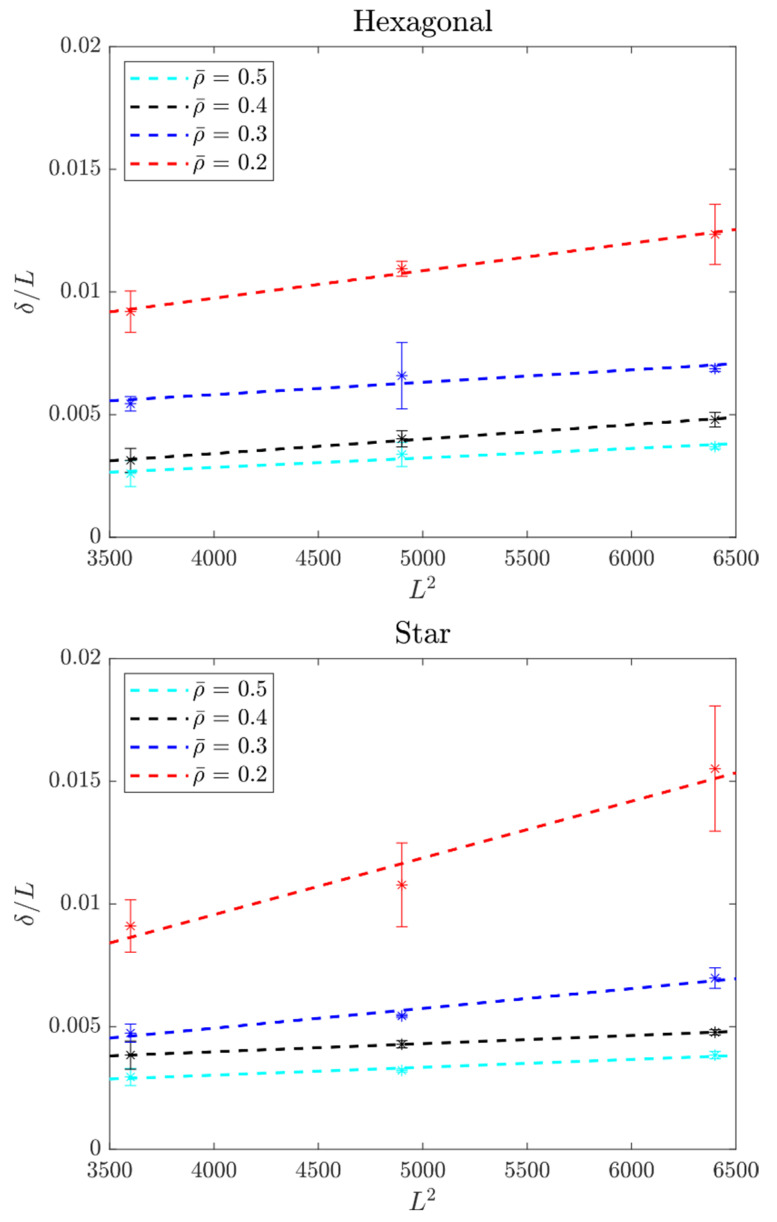
*δ/L* vs. *L*^2^ for both lattice structures.

From the force-deflection curves, the data for *δ/L* at *F* = 4 N are summarized in Table 2 and Table 3. Then they were re-plotted for all samples in a *δ/L* vs. *L*^2^ graph, as shown in Figure 6. Note from the results presented in Table 2 and Table 3, that the samples became stiffer via two paths: (i) increasing the infill density and (ii) reducing the distance between supports. For the former, more infill density resulted in a higher amount of material used within the sample. This material, in turn, participated in withstanding the external load. For the latter, recall that the elastic transverse deflection scales cubically with the length. Finally, higher variability was observed for samples with higher porosity, as these were more sensitive to defects at the characteristic lattice dimension. The higher the slope, the softer the structure. In contrast, those lines with lower slopes corresponded to stiffer structures. The intercept represented shear-deflections, in this case caused by the stiffness of the stacking of layers and not the in-plane shear modulus of the lattices.

### 3.2. Flexural Elasticity of Lattice Beams

**Figure 7:**
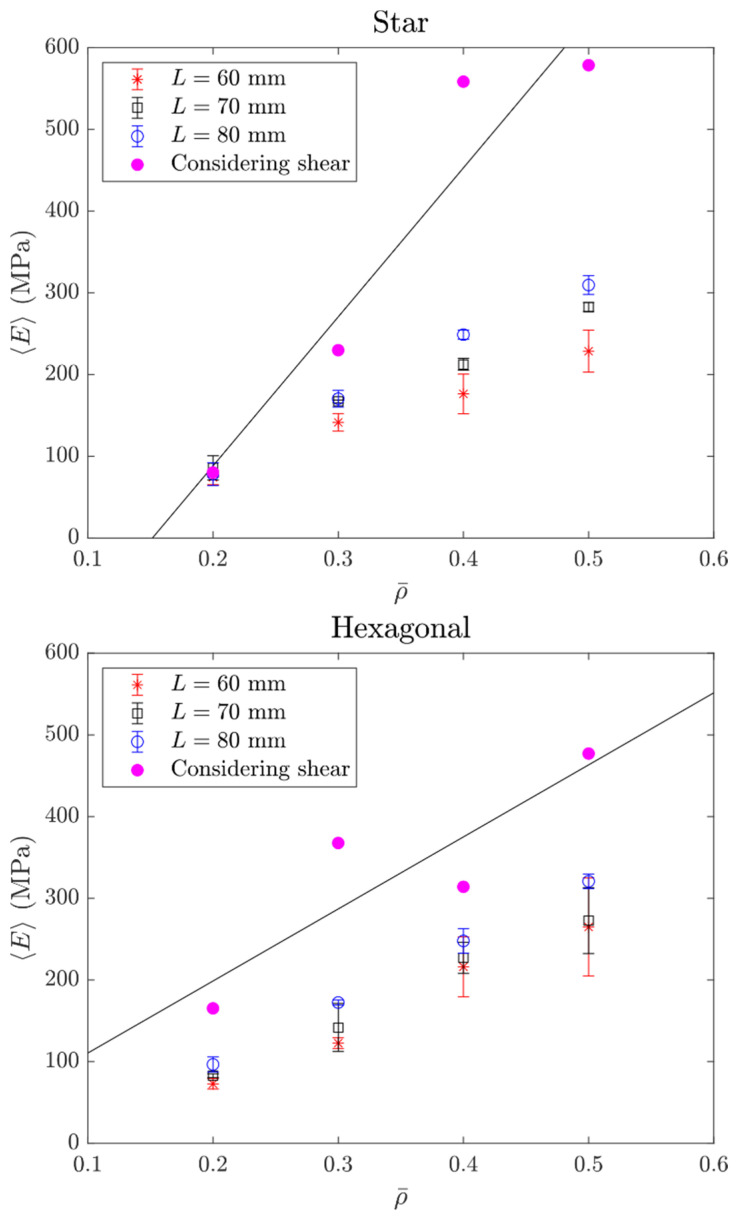
Effective Young’s modulus vs. relative (infill density).

The slopes in Figure 6 were used to obtain the effective Young’s modulus 〈E〉. These results were plotted as a function of the infill density $\bar{\rho }$ in Figure 7. Note from Figure 7 that 〈E〉~$\bar{\rho }$ follows a slightly linear trend. In contrast, the effective shear did not follow a clear trend because, in this case, the shear was that of the thickness of the samples; therefore, it was not included in Figure 7. Samples fabricated here had some discontinuities at their edge that affected the shear in bending; this will be discussed in Section 4.

### 3.3. Shear Sensitivity in the Transverse Deflection of Lattice Beams

If we now take Equation (1) and the no-shear version of it, we can work out an expression for the ratio in between them. This ratio can be used to check where these two differ and is obtained as

(4) $$1-\left(\frac{FL}{4\kappa GA}\right)\left(\frac{1}{\delta }\right).$$

From further inspection of Equation (4), we note that in the lattice beams tested here, the transverse deflection of beams without considering shear can lead to misleading results. Note that if the deflection does not consider shear, the Young’s modulus can be divided by the factor given by Equation (4) to get the shear-inclusive deflection. The differences in the effective Young’s modulus are shown in Figure 7.

The effective stiffness values considering shear were larger because the experimental deflection value was divided into bending deformation (slope) and shear deformation (intercept). Using a longer separation L between supports and directly calculating the effective stiffness from here yielded results that were closer to the value considering shear. For example, in the hexagonal topology with a 20% infill, the deviation was 55.9% with respect to the shear-inclusive model for *L* = 60 mm, while it was 41.6% for *L* = 80 mm. Therefore, and given that the separations *L* were small relative to the depth, shear deformation must be included in the calculation of the effective stiffness of lattice beams. The effect of the topology was also noticeable, having a trend similar to a function of the density. However, significant variations were observed, which were attributed to the defects at the lattice characteristic level. Also, low density lattices were more sensitive to edge effects, as it will discussed in the following section. Note that despite theoretically using the same amount of material, the hexagonal topology at 20% infill had twice the effective stiffness of the star topology at the same percentage. At a 30% infill, the hexagonal arrangement was 60% stiffer. This changed at larger infill percentages, where the star arrangement was now the stiffest. At 40%, the star arrangement was 78% stiffer, while at 50% infill, the star arrangement had 21% more stiffness than the hexagonal topology.

### 3.4. Micro-Characterization of PLA-Wood Lattices

**Figure 8:**
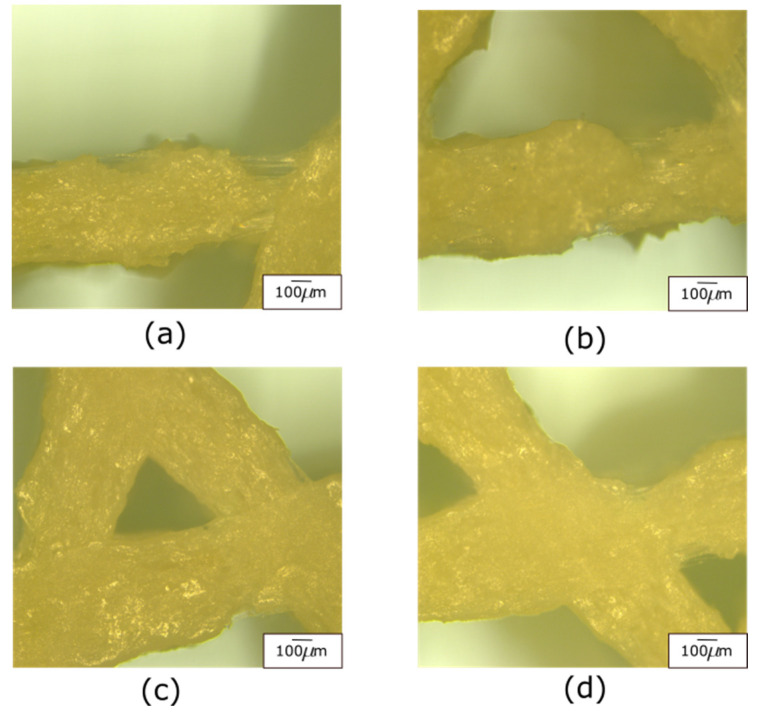
Micro-characterization of PLA-wood lattices. Representative optical micrographs with 50× magnification and 100 µm of measurement scale exhibiting defects in star topology (**a**,**b**) 40% and (**c**,**d**) 20% infill density. All micrographs correspond to the bottom side of the samples.

The variability obtained in the results presented in Figure 5 and Figure 6 prompted further characterization using micrographs. The results from the micro-characterization revealed a high number of defects at the raster level (Figure 8). The defects and the variation in the diameters of the filaments produced a lack of homogeneity, affecting the mechanical properties. Some areas had low adhesion between the matrix and the reinforcement, causing delamination of the fibers and thinning of the matrix, which looked like the fibers were being ripped from the PLA-matrix. The areas that presented this type of phenomenon became susceptible to stress concentrators, hence mechanical failure of the structure. The lack of an appropriate bonding between the PLA matrix and wood fibers also resulted in a poor level of repeatability (Figure 8). Even though defects were more frequently encountered in high porosity samples, these were present in both topologies and in the different infill densities. Figure 8 only includes micrographs for the star topology, as these defects were more visually evident.

## 4. Discussion

Results here are restricted to the orientation shown in Figure 3. Parts fabricated with FDM were anisotropic. The effect of fabrication orientation was studied for fully-dense PLA-wood samples in [41]. Composites are known to be anisotropic, as their properties depend on the orientation of the reinforcement fibers. The PLA-wood used here was in turn anisotropic. Nevertheless, to avoid this property having a significant influence on the mechanical properties of the lattices, all the samples were fabricated with the same orientation with respect to the printing plane. This resulted in rasters conforming the lattice being oriented at the same angles in all the samples of the same topology—regardless of the infill density (as the infill density does not affect the rasters orientations). On the other hand, the lattice anisotropy was a different story that will involve further study as the isotropy of 2D patterns depends on the rotational symmetry of the lattice [42,43]. The effective properties of the infill lattices tested here were also in-plane directionally dependent, but we restricted the study to varying only their infill density. From inspecting Figure 6 and Figure 7, a clear dependence of the effective Young’s modulus on the infill density was observed.

The trend observed 〈E〉~$\bar{\rho }$ appeared to follow a linear relation for the range of infill densities tested. However, it was shown that the effective elasticity of these lattices had a nonlinear relationship to the relative density (which is related to the infill percentage) [44,45]. For the range tested here, 20–50% resembled a straight line. We were aware that this structure had anisotropy also for the in-plane properties, and that the orientation analyzed in [44,45] was different from the one tested here. However, the properties in other directions also had a nonlinear relationship with the relative density. Still, for the range tested here, this appeared to be linear.

**Figure 9:**
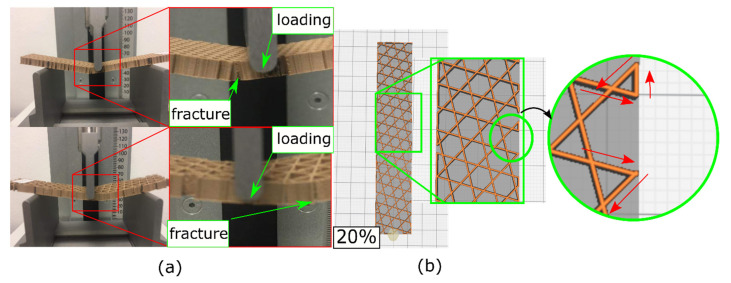
Failure of the lattice PLA-wood samples (**a**); note the failure of being slightly off the loading point, owing to the open zones exposed in (**b**).

Failure of the samples under 3-point bending occurred at locations slightly off the mid-point (Figure 9a). This was attributed to the edges of the samples as they had open zones, more prone to cracks and failure. These open zones were generated by the trajectory of the nozzle, coming from one side of the sample, then reaching the other side, moving along the edge, and returning (see Figure 9b where they are represented with red arrows). This suggested that modifying the nozzle trajectory could lead to different results for failure.

Regarding the material, several fabrication characteristics might affect the mechanical properties of PLA-wood composites. The mechanical behavior of a composite material depends on the location and composition of both fibers and matrix, as well as the interfacial interactions linking them. While the wood has a polar-hydrophilic surface, PLA has a non-polar hydrophobic one, hindering the attaching [46]. A lack of synergy is ineffective for load transmission to the fibers. This causes a degradation of the observed mechanical properties of PLA with wood fibers when compared to those of pure PLA [47,48].

A larger fiber content results in a reduction of the mechanical properties due to stress accumulation and greater shearing intensity. When the loading of wood fibers is low, it is uniformly distributed in the PLA matrix, causing an absorption of the external loading force. If the size of the fibers is too small, they are prone to forming aggregates, which become stress concentrators in the matrix that diminish the mechanical properties of the structure. When the agglomerated fibers are not evenly distributed, layers are detached under external force, forming holes [49,50]. Thus, under an external load, the combination of these characteristics might result in the formation of voids and failure because the particle size interfacial adhesion would be determined by the small surface energy of the large wood particles [49,51,52]. When wood fibers are added to PLA the surface of the polymer will became more porous. The porosity may affect the mechanical properties of polymer-based composites parts produced by FDM [53]. PLA-wood is recommended for applications under compression instead of tension [54]. As recently reported for these composites, infill density is the most influential parameter of the tensile strength, followed by building orientation and layer height [23]. However, when other parameters are varied, defects at the raster level tend to be obscured. These defects become more relevant and influential in lattice-type samples.

## 5. Conclusions

The stiffness of FDM samples with two different infill lattice patterns was characterized via 3-point bending. The samples were fabricated using PLA-wood. Several aspects that influenced the stiffness of lattice beams were revealed in the mechanical and micro-characterization. The infill relative density had a significant impact on the effective mechanical properties. The topology also had an influence on the effective stiffness; despite using the same amount of material, the hexagonal topology was 100 to 60% stiffer than the star topology at low infill percentages. However, at larger infill percentages, the star arrangement was 78 to 21% stiffer than the hexagonal topology. In addition to this, lattice beams were more sensitive to shear deformations; the contribution of shear was thus evaluated, and the shear-inclusive model produced larger values for the effective stiffness. Additionally, the extrusion paths can lead to different failure mechanisms; this is another reason why, despite having the same infill density, PLA-wood samples with different topologies had different values of effective stiffness and high variability.

Despite PLA-wood being an option that offers the reduction of synthetic polymers used in plenty of applications, the composite material interaction and its fabrication process can lead to the printing of structures with defects, compromising the prediction of their mechanical properties. Observing the effect of different infills used for the same additive manufacturing process will lead to a better choice of infill patterns depending on the final application of the 3D-printed component. The defects and properties of the material must also be considered, focusing on patterns and densities with a higher repeatability. The results presented here corresponded only to the static loading scenario; if this novel material is used in any engineering application further characterization methods are demanded. For instance, the response under dynamic loading is likely to be affected by the defects and lattice topologies. Other loading scenarios that are needed before using PLA-wood lattices in any application could include torsional and modal (vibrations).

Organic designs very often are restricted to geometrical aspects; with the inclusion of natural materials in 3D printing such as wood, other applications scenarios can be explored. Prior this, a proper characterization of the mechanical properties of structures with wood particle inclusions is demanded. Other porous structures could be fabricated and then characterized, including structures with a closer resemblance to those encountered in nature with random and functionally graded porosity.

## Figures and Tables

**Table 1:** Parameters for experimental work.

Constants		Variables	
Layer height (mm)	0.1	$\bar{\rho }$ (%)	20, 30, 40, 50
Extruder and bed temperature (°C)	19,060	*L* (mm)	60, 70, 80
Print speed (mm/s)	30	Infill topologies	Hexagonal, Star

**Table 2:** *δ/L* data for the star structure.

$\bar{\rho }$ (%)		*L* (mm)	
	60	70	80
50	0.00294 ± 0.00034 ^c,A^	0.00321 ± 0.00007 ^b,A^	0.00383 ± 0.00014 ^b,B^
40	0.00384 ± 0.00057 ^bc,A^	0.00428 ± 0.00015 ^b,AB^	0.00477 ± 0.00010 ^b,B^
30	0.00474 ± 0.00038 ^b,A^	0.00544 ± 0.00006 ^b,A^	0.00698 ± 0.00042 ^b,B^
20	0.00910 ± 0.00107 ^a,A^	0.01078 ± 0.00171 ^a,A^	0.01551 ± 0.00255 ^a,B^

Mean value ± SD. Values within the same column followed by different lowercase letters are significantly different (*p* < 0.05); values within the same line followed by different capital letters are significantly different (*p* < 0.05).

**Table 3:** *δ/L* data for the hexagon structure.

$\bar{\rho }$ (%)		*L* (mm)	
	60	70	80
50	0.00260 ± 0.00053 ^c,A^	0.00338 ± 0.00049 ^c,AB^	0.00370 ± 0.00010 ^c,B^
40	0.00314 ± 0.00049 ^c,A^	0.00402 ± 0.00033 ^c,AB^	0.00480 ± 0.00030 ^c,B^
30	0.00544 ± 0.00029 ^b,A^	0.00659 ± 0.00135 ^b,A^	0.00688 ± 0.00013 ^b,A^
20	0.00920 ± 0.00084 ^a,A^	0.01094 ± 0.00031 ^a,AB^	0.01235 ± 0.00123 ^a,B^

Mean value ± SD. Values within the same column followed by different lowercase letters are significantly different (*p* < 0.05); values within the same line followed by different capital letters are significantly different (*p* < 0.05).

## Data Availability

Data is contained within the article.
